# Evaluation of a national multidisciplinary meeting for non-tuberculous mycobacterial disease

**DOI:** 10.5588/ijtldopen.24.0044

**Published:** 2024-06-01

**Authors:** A. Lemson, T.A. Koster, N. Carpaij, C. Magis-Escurra, M. Boeree, R. Stemkens, R.E. Aarnoutse, A. van Laarhoven, R. van Crevel, J. van Ingen, W. Hoefsloot

**Affiliations:** ^1^ Department of Pulmonary Diseases, Research Institute for Medical Innovation, Radboud University Medical Centre, Nijmegen, The Netherlands;; ^2^ Department of Pharmacy, Research Institute for Medical Innovation, Radboud University Medical Centre, Nijmegen, The Netherlands;; ^3^ Department of Internal Medicine and Radboud Center for Infectious Diseases, Radboud University Medical Centre, Nijmegen, the Netherlands;; ^4^ Department of Medical Microbiology, Research Institute for Medical Innovation, Radboud University Medical Centre, Nijmegen, The Netherlands

**Keywords:** *Mycobacterium avium* complex, *Mycobacterium abscessus*, NTM-PD, EP-NTM

Dear Editor,

Non-tuberculous mycobacteria (NTM) are opportunistic pathogens that mostly cause pulmonary disease (NTM-PD), but also extrapulmonary (EP-NTM) skin and soft tissue infections, lymphadenitis, and in severely immunocompromised people can result in disseminated infections.^1^ In the Netherlands, the annual prevalence of NTM-PD is estimated to be between 2.3 and 4.5 per 100,000 persons.^2^ Diagnosing NTM disease is complex because of nonspecific symptoms, the influence of underlying diseases and the lack of specific diagnostic tools. Antimycobacterial treatment is also complicated because of drug resistance, the need for prolonged multidrug regimens, frequent adverse drug reactions and drug–drug interactions.^3,4^ International guidelines provide only limited guidance on managing these complications.^4^ Because of the complex nature of NTM disease – as well as the multimorbidity and polypharmacy of patients they occur in – multiple specialists are often consulted on a single case.^5^ In 2018, to optimise care for patients with NTM disease, we established a 1-h, bi-weekly multidisciplinary meeting, open to physicians throughout the Netherlands and other Dutch-speaking regions. These hybrid meetings were attended by specialised pulmonologists, infectious disease specialists, clinical microbiologists, and hospital pharmacists. Cases were presented by the consulting physician, after which the specialists systematically provided input. Pulmonologists and infectious disease specialists oversaw NTM-PD and EP-NTM cases, respectively. Clinical microbiologists evaluated the isolated NTM species, drug susceptibility, composition of and response to antimycobacterial treatment. Hospital pharmacists assessed the dosing strategies, drug-drug interactions and outcomes of therapeutic drug monitoring. We evaluated the clinical cases discussed during our multidisciplinary meetings from 2019 to 2020 and present an overview of patient characteristics, frequency, and nature of the consultations and recommendations given. The data were pseudonymised and collected in an SPSS database (IBM SPSS Statistics v27; IBM, Armonk, NY, USA) for descriptive statistics. A waiver of informed consent was obtained from the research ethics committee.

Thirty-eight meetings were held over this period, during which 120 cases in 91 individual patients with (presumed) NTM disease were discussed. On average, 3.2 patients were discussed per meeting. The number of consultations increased over the study period (42 cases in 2019; and 78 cases in 2020), mostly due to an increase in consultations from external physicians (31% to 40%, respectively). An overview of patient characteristics, nature of consultations received, and recommendations given per disease manifestation is presented in the Table. Most patients had (presumed) NTM-PD (62/91, 68.1%). A total of 26/91 (28.6%) patients with (presumed) EP-NTM manifestations were discussed, of which 10/91 (11%) had skin and/or soft tissue infections and 6/91 (6.6%) had lymph node infections. Seven out of 91 (7.7%) patients had disseminated NTM disease. The majority of cases had *M. avium* complex or *M. abscessus* disease (see Table).

**Table.** Patient characteristics, consultation pattern and meeting outcomes.*

	Pulmonary NTM *n* (%)	Extrapulmonary NTM *n* (%)	Total *n* (%)
Patients	62 (68.1)	26 (28.6)	91 (100)^†^
Mean age at first meeting, years	57.1	54.9	55.9
Sex			
Male	30 (48.4)	11 (42.3)	43 (47.3)
Female	32 (51.6)	15 (57.7)	48 (52.7)
Patient history			
Previous mycobacterial infection	19 (30.6)	8 (30.8)	27 (29.7)
Previous antimycobacterial treatment	14 (22.6)	7 (26.9)	21 (23.1)
Current NTM treatment	27 (43.5)	14 (53.8)	41 (45.1)
Chronic pulmonary disease	42 (67.7)	2 (7.7)	44 (48.4)
Immunodeficiency	5 (8.1)	3 (11.5)	8 (8.8)
Immunosuppression	25 (40.3)	9 (34.6)	34 (37.4)
Number of consultations per patient			
1	52 (83.9)	17 (65.4)	72 (79.1)
>1	10 (16.1)	9 (34.6)	19 (20.9)
Mean number of consultations per patient	1.3	1.6	1.3
Cases	79 (65.8)	41 (34.2)	120 (100)
Mycobacterium species			
*M. avium* complex	49 (62.0)	11 (26.8)	60 (50.0)
*M. abscessus*	9 (11.4)	11 (26.8)	20 (16.7)
*M. chelonae*	4 (5.1)	5 (12.2)	9 (7.5)
*M. xenopi*	4 (5.1)	1 (2.4)	5 (4.2)
*M. marinum*	0 (0)	5 (12.2)	5 (4.2)
*M. kansasii*	4 (5.1)	0 (0)	4 (3.3)
Other	4 (5.1)	6 (14.6)	10 (8.3)
No positive culture	2 (2.5)	2 (4.9)	7 (5.8)
Nature of consultation^‡^			
Recommended treatment regimen	31 (39.2)	16 (39)	47 (39.1)
Clinical relevance of isolated NTM species	27 (34.2)	12 (29.3)	40 (33.3)
Not specified	12 (15.2)	9 (22)	21 (17.5)
Diagnostics	8 (10.1)	5 (12.2)	15 (12.5)
Surgery indication	7 (8.9)	6 (14.6)	13 (10.8)
Progression under treatment	5 (6.3)	4 (9.8)	9 (7.5)
Predisposing condition or event	2 (2.5)	0 (0)	2 (1.7)
Recommendation			
Additional diagnostics	49 (62.0)	25 (61.0)	76 (63.3)
Supportive care	18 (22.8)	7 (17.1)	26 (21.7)
Start hypertonic saline inhalation	37 (46.8)	—	—
Start antimycobacterial treatment	16 (20.3)	5 (12.2)	22 (18.3)
Continue antimycobacterial treatment	23 (29.1)	21 (51.2)	44 (36.7)
Adjust antimycobacterial treatment	10 (12.7)	5 (12.2)	15 (12.5)
Stop antimycobacterial treatment	4 (5.1)	2 (4.9)	6 (5.0)
Surgical intervention	7 (8.9)	10 (24.4)	17 (14.2)
Admission to Radboudumc	8 (10.1)	1 (2.4)	9 (7.5)
In line with the NTM-PD guidelines^§^	57 (72.1)	34 (82.9)	93 (77.5)

The number (*N*) of patients and cases were separated due to different reasons for consultations and recommendations between primary and re-consultation. ^†^Disease manifestation was not recorded for 3 patients. ^‡^30 consultations included two topics. ^§^The 2020 NTM-PD guideline was also adhered to for patients with extrapulmonary NTM, where applicable.

NTM = non-tuberculous mycobacteria; Radboudumc = Radboud University Medical Centre; NTM-PD = NTM causing pulmonary disease.

The complexity of diagnosis and management of NTM disease, and thus the need for multidisciplinary consultations, is reflected by the increasing number and diverse nature of consultations by physicians in the Netherlands. Most patients presented only once to the multidisciplinary meeting (*n* = 72, 79%); re-consultation was more frequent for EP-NTM than for NTM-PD patients (34.6% vs. 16.1%). This consultation pattern may be explained by the lack of guidelines for EP-NTM, the fewer options to evaluate treatment outcome and the higher percentage of EP-NTM vs. NTM-PD patients on antimycobacterial treatment, requiring more frequent follow-up. The most common consultation was for advice regarding (initiation of) antimycobacterial treatment (39%). Sixty-five out of 120 (54.2%) cases were receiving antimycobacterial treatment at the time of discussion, but required modification (*n* = 15, 23.1%), cessation (*n* = 6, 9.2%), or admission to the reference clinic (*n* = 9, 7.5%) at Radboudumc, Nijmegen, The Netherlands. These high proportions may reflect limited experience outside of expert centres,^6,7^ but are more likely a result of the common complications associated with NTM treatment, such as adverse drug reactions, drug–drug interactions or suboptimal treatment effects, all of which require treatment adjustments.

The multidisciplinary meeting resulted in a high number of active recommendations for both NTM-PD and EP-NTM cases. Although most cases were presented with a specific question, recommendations extended the primary nature of consultation. This finding strengthens our conviction that care for patients with NTM disease requires a team effort. We argue that all patients with (a clinical suspicion of) NTM disease should be discussed by a multidisciplinary team of experts, as experience of NTM disease is often limited and it assists in optimising diagnosis and treatment, and streamlines access to clinical trials. Antimycobacterial treatment often requires modification; however, alternative treatment options are scarce and guideline recommendations for treatment complications are limited.^8^ Therefore, we advocate for a repeated consultation with a multidisciplinary team after 3–6 months of antimycobacterial treatment, timed to coincide with the expected occurrence of culture conversion.^9^

Although *M. abscessus* disease is quite rare in the Netherlands,^10^ it represented 16.7% of the cases in our evaluation. This considerable proportion is unsurprising because of its progressive nature, and fact that treatment is often ineffective and toxic.^4^ Another interesting finding is the high rate of recurrent NTM disease with comparable numbers between NTM-PD and EP-NTM patients in our study population. Because recurrent NTM disease requires additional diagnostics, such as drug susceptibility testing and therapeutic drug monitoring, and has implications for antimycobacterial treatment,^4^ we encourage discussion by a multidisciplinary team.

This evaluation has several limitations. First, the retrospective nature of this study limits the amount of information available to that in patient files. To systematically collect data, we introduced a form with pre-set questions and fields to record essential information in the electronic medical file in July 2019. Second, the study population is not fully representative of NTM disease patients in the Netherlands, and the need to discuss with a multidisciplinary team introduces substantial selection bias. Moreover, not all physicians were aware of the existence of our multidisciplinary meetings during the study period. Third, the recommendations by the multidisciplinary team are in part driven by local expertise. For example, we previously reported the importance of hypertonic saline inhalation in patients with NTM-PD^11^ for sputum clearance and in vitro antimycobacterial effects. This is reflected in the number of recommendations in this study to initiate hypertonic saline inhalation for NTM-PD. Finally, the evaluation covers the start-up period of the multidisciplinary meetings and the volume of consultations has continued to grow over recent years. For example, although not included in this analysis, 257 new patients were discussed in the multidisciplinary meetings between 2021–2023, resulting in an estimated average of 4.5 newly consulted patients per meeting.

Based on this evaluation, we advocate for 1) establishing regional and/or national multidisciplinary expert teams for NTM disease, including pulmonologists, infectious disease specialists, clinical microbiologists and hospital pharmacists; and 2) consultation with the multidisciplinary team for patients with (a clinical suspicion of) NTM disease and re-consultation 3–6 months after the start of antimycobacterial treatment.
